# The nuclear localization signal-mediated nuclear targeting of herpes simplex virus 1 early protein UL2 is important for efficient viral production

**DOI:** 10.18632/aging.102786

**Published:** 2020-02-07

**Authors:** Meili Li, Xingmei Zou, Yuanfang Wang, Zuo Xu, Xiaowen Ou, Yiwen Li, Delong Liu, Yingjie Guo, Yangxi Deng, Si Jiang, Tong Li, Shaoxuan Shi, Yilong Bao, Tao Peng, Mingsheng Cai

**Affiliations:** 1 Guangdong Provincial Key Laboratory of Allergy and Clinical Immunology, Second Affiliated Hospital of Guangzhou Medical University, The Sixth Affiliated Hospital of Guangzhou Medical University, Qingyuan People’s Hospital, Sino-French Hoffmann Institute, School of Basic Medical Science, Guangzhou Medical University, Guangzhou 510260, Guangdong, China; 2 State Key Laboratory of Respiratory Diseases, Sino-French Hoffmann Institute, School of Basic Medical Science, Guangzhou Medical University, Panyu, Guangzhou 511436, Guangdong, China; 3 South China Vaccine Corporation Limited, Guangzhou Science Park, Guangzhou 510663, Guangdong, China

**Keywords:** HSV-1 UL2, nuclear localization signal, recombinant virus

## Abstract

Herpes simplex virus 1 (HSV-1) is a representative alphaherpesvirus that can provoke a series of severe diseases to human being, but its exact pathogenesis is not perfectly understood. UL2, a uracil-DNA glycosylase involved in the process of HSV-1 DNA replication, has been shown to be predominantly targeted to the nuclei in our previous study, yet little is established regarding the subcellular localization signal or its related function of UL2 during HSV-1 propagation. Here, by creating a number of UL2 variants merged with enhanced yellow fluorescent protein, an authentic nuclear localization signal (NLS) of UL2 was, for the first time, identified and profiled to amino acids (aa) 1 to 17 (MKRACSRSPSPRRRPSS), and ^12^RRR^14^ was indispensable for its nuclear accumulation. Besides, the predicted nuclear export signal (aa 225 to 240) of UL2 was determined to be nonfunctional. Based on the HSV-1 bacterial artificial chromosome and homologous recombination technique, three recombinant viruses with mutations of the identified NLS, deletion and revertant of UL2 were constructed to assess the effect of UL2 nuclear targeting on HSV-1 replication. Compared to the wild type HSV-1, UL2 deletion remarkably restrained viral production, and mutation of NLS targeting UL2 to cytoplasm (pan-cellular distribution) in recombinant virus-infected cells showed a certain degree of deficiency in HSV-1 proliferation. Moreover, recombinant virus with UL2 deletion exhibited serious damages of viral DNA synthesis and mRNA expression, and these processes were partially disrupted in the recombinant virus with UL2 NLS mutation. Collectively, we had established a functional NLS in UL2 and showed that the NLS-mediated nuclear translocation of UL2 was important for efficient production of HSV-1. These data were of significance for further clarifying the biological function of UL2 during HSV-1 infection.

## INTRODUCTION

Herpes simplex virus 1 (HSV-1), a epidemic human pathogen with a high ratio of infection in the population, can cause a number of diseases that is extremely adverse to public health. Upon infection, HSV-1 can trigger ulcers in mouth or lips, genital herpes, encephalitis and keratitis [1, 2], then establish latent infection in trigeminal ganglion. However, the latent virus can be re-activated to induce lytic infection, which leads to the occurrence of various diseases [3]. Although acyclovir and other related drugs are efficient anti-viral drugs developed against HSV-1 infection, the exact pathogenesis of HSV-1 is still unclear.

UL2 protein, the gene product of *UL2*, has been reported as a uracil-DNA glycosylase (UDG) [4]. UDG is demonstrated to be associated with the DNA excision repair pathway, which precisely cuts the inaccurate uracil from the synthetic DNA [5]. UDG also participates in the viral replisome formation, through its combination with viral DNA polymerase [6]. Furthermore, UDG may be essential for HSV-1 reactivation, since the UL2 mutant virus shows reduced neurovirulence and decreased recurrent infection from latency [7]. Consequently, UL2 is a versatile protein. It’s shown that one of the homologues of HSV-1 UL2, human cytomegalovirus (HCMV) UL114 protein, is required for viral DNA replication [8], which functions in cooperation with UL44 (HCMV processivity factor) and UL54 (HCMV DNA polymerase) [9, 10]. In addition, BKRF3 also can enhance Epstein-Barr virus (EBV) oriLyt-initiated plasmid replication [11–13]. Our previous study demonstrated that in live cells, HSV-1 UL2 is almost absolutely targeted to the nucleus without the presence of other viral components [14, 15], yet little is established about its functional localization motif(s). Therefore, this is unquestionably of interest and impel us to investigate its subcellular localization signals, as well as their functions in the course of HSV-1 infection.

In the present study, live cells fluorescence microscopy technique, which is extensively adapted and utilized in our lab [15–24], was exploited to identify the functional domains of UL2. By sequence analysis and constructing a large number of deletion mutants of UL2 fused with green fluorescent protein variant enhanced yellow fluorescent protein (EYFP), the functional nuclear localization signal (NLS) of UL2 was characterized, but its potential nuclear export signal (NES) was identified to be nonfunctional. Based on HSV-1 bacterial artificial chromosome (BAC) and homologous recombination technique, recombinant viruses with mutation of the NLS, deletion and revertant of UL2 were constructed, and results showed that the NLS-mediated nuclear trafficking of UL2 was important for the efficient yield of HSV-1. Together, these data are crucial for further discerning UL2-mediated biological function in HSV-1 life cycle.

## RESULTS

### Preliminary identification of the NLS and NES regions of UL2

**Figure 1:**
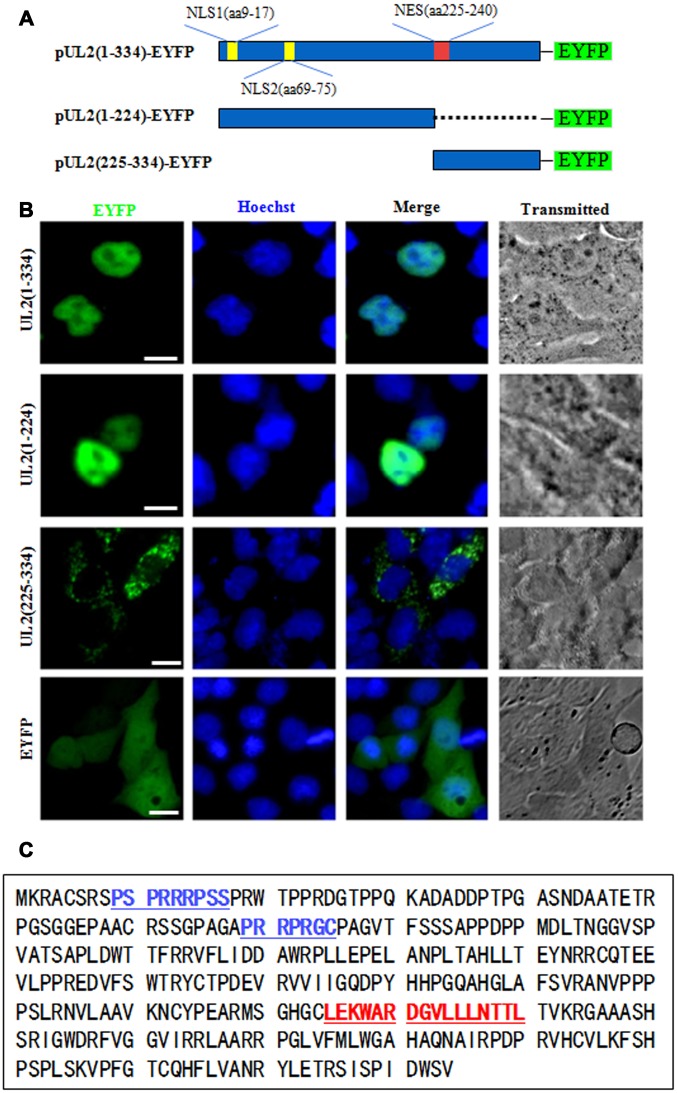
**Preliminary determination of the NLS and NES regions of UL2.** (**A**) Schematic diagram of full-length UL2(1-334), UL2(1-224) and UL2(225-334) fused with an EYFP monomer at its N terminus. (**B**) Subcellular localization of UL2(1-334)-EYFP, UL2(1-224)-EYFP, UL2(225-334)-EYFP and EYFP in live COS-7 cells. (**C**) Bioinformatics analysis of potential NLS and NES of UL2. Blue color labelled residues represent potential NLS1 and NLS2. Red color labelled residues represent potential NES. All scale bars indicate 10 μm.

The functional implementation of a specific protein is highly correlated with its subcelular distribution. It’s generally believed that almost every protein possesses a definite subcellular transport signal, and diverse subcellular localizations probably represent distinct functions of a particular viral protein [25, 26]. To figure out the NLS region of UL2, amino acids (aa) 1 to 334 of full-length UL2 was firstly cut into two sections aa 1 to 224 and aa 225 to 334, since the former section is rich in basic aa, whereas the latter is rich in hydrophobic aa. Then, these two segments were fused to the N-terminus of EYFP (Figure 1A), and the related constructs were transfected into COS-7 cells for the detection of their subcellular localizations. As shown in Figure 1B, aa1-224-EYFP was located exclusively in the nucleus, but not the nucleolus, which was similar to the subcellular distribution pattern of full-length UL2 (UL2-EYFP). Instead, aa225-334-EYFP showed pan-cytoplasmic localization. As negative control, the fluorescence of EYFP vector was evenly distributed throughout the cells without nucleolus. Therefore, these results clearly showed that aa 1-224 and 225-334 were necessary for the nuclear accumulation and cytoplasmic localization of UL2, respectively, and they may contain functional NLS and NES. It is well known that the NLS is generally composed of basic residues [27]. From the above results we speculated that there was a nuclear accumulation or direct signal in UL2. Bioinformatics analysis using PSORT II [28] predicted that there are two potential NLSs in the arginine-rich regions (Figure 1C, blue color letters), namely PSPRRRPSS at aa 9 to 17 and PRRPRGC at aa 69 to 75, which were denominated as potential NLS1 and NLS2, respectively.

### Characterization of the functional NLS and its key aa of UL2

**Figure 2:**
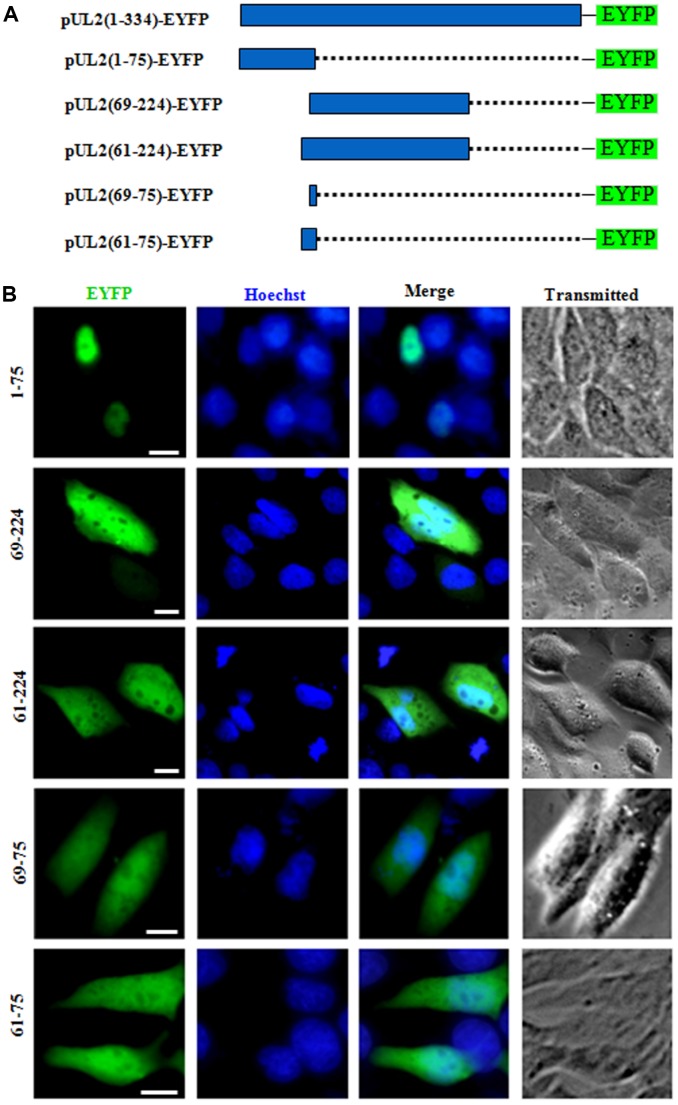
**The predicted NLS2 of UL2 is nonfunctional.** (**A**) Schematic representation of WT UL2 and its deletion mutants UL2(1-75), UL2(69-224), UL2(61-224), UL2(69-75) and UL2(61-75) fused with EYFP. (**B**) Subcellular localization of these UL2 deletion mutants (shown in **A**) in plasmid-transfected live COS-7 cells. All scale bars indicate 10 μm.

To determine whether one or both of the predicted NLSs are functional, two deletion mutants aa1-75-EYFP and aa69-224-EYFP were constructed (Figure 2A), which were then transfected into COS-7 cells to analyze their subcellular localizations. As results, aa1-75-EYFP showed predominantly nuclear localization, while aa69-224-EYFP exhibited pan-cellular distribution (Figure 2B), indicating aa 1 to 75 encompasses functional NLS, and the predicted NLS2 may be nonfunctional. To verify this hypothesis, aa 69 to 224 was extended to aa 61 to 224 and fused with EYFP (aa61-224-EYFP), and aa69-75-EYFP was also constructed (Figure 2A). Results showed that the subcellular localization patterns of aa61-224-EYFP and aa69-75-EYFP were identical to that of aa69-224-EYFP (Figure 2B), suggesting that the predicted NLS2 is nonfunctional. In addition, when the predicted NLS2 was extended to aa 61 to 75 and fused with EYFP (aa61-75-EYFP) (Figure 2A), its fluorescence showed similar subcellular distribution with that of aa69-75-EYFP (Figure 2B), further confirming the predicted NLS2 is not a functional NLS.

**Figure 3:**
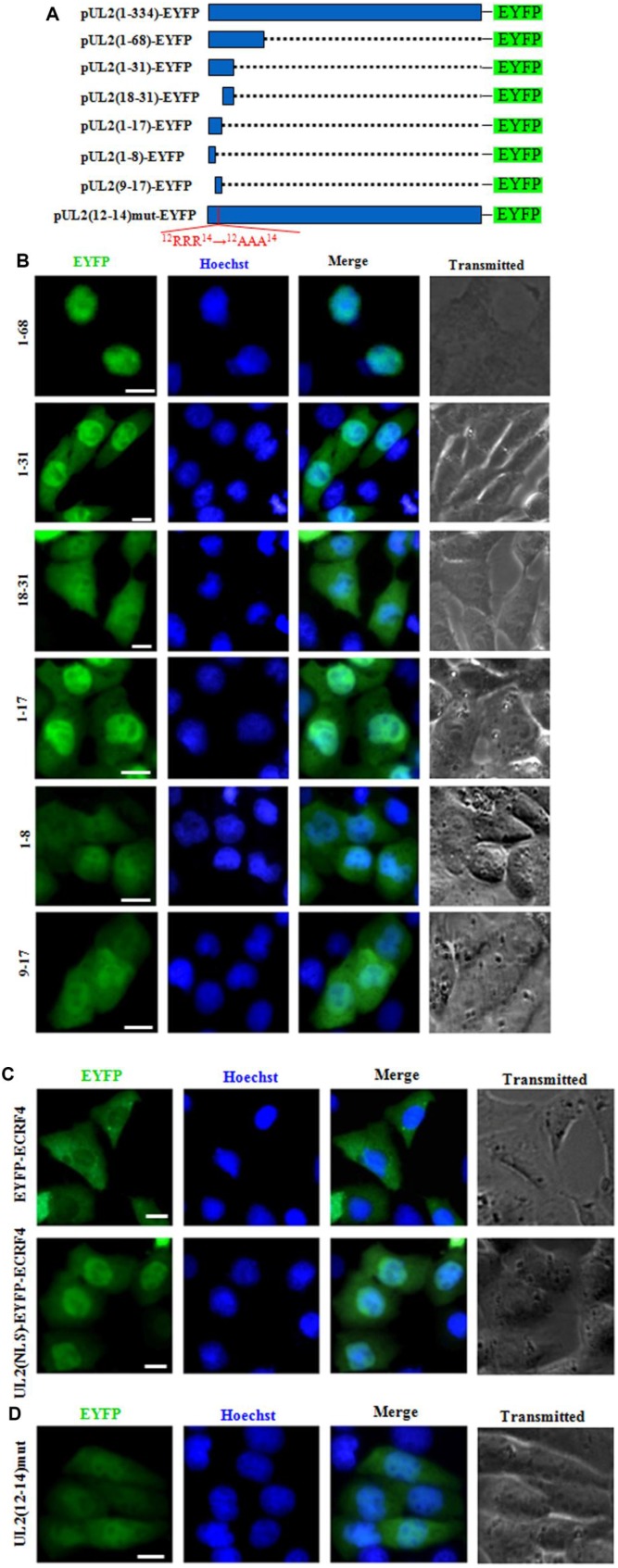
**Characterization of the functional NLS and its key aa of UL2.** (**A**) Schematic representation of WT UL2 and its deletion mutants UL2(1-68), UL2(1-31), UL2(18-31), UL2(1-17), UL2(1-8), UL2(9-17) and UL2(12-14)mut fused with EYFP. (**B**–**D**) Subcellular localization of these UL2 deletion mutants (**B** and **D**, shown in **A**), EYFP-ECRF4, or UL2(NLS)-EYFP-ECRF4 (**C**) in plasmid-transfected live COS-7 cells. All scale bars indicate 10 μm.

To further explore the functional NLS of UL2, aa 1 to 68 that does not contains the predicted NLS2 was fused with EYFP (aa1-68-EYFP) (Figure 3A) and tested in COS-7 cells. As shown in Figure 3B, aa1-68-EYFP also showed similar subcellular localization with that of aa1-224-EYFP, further proving the supposed NLS2 is nonfunctional, and aa 1 to 68 contains functional NLS. Besides, aa1-31-EYFP was also constructed (Figure 3A) and its fluorescence in nucleus was evidently more than that of the cytoplasm (Figure 3B), indicating this region possesses functional NLS.

To continue identify the exact NLS region of UL2, aa 1 to 31 was divided into two fragments according to the predicted region of potential NLS1, namely aa 1 to 17 and aa 18 to 31, then they were fused with EYFP (aa1-17-EYFP and aa18-31-EYFP) (Figure 3A) and transfected into COS-7 cells. As shown in Figure 3B, aa1-17-EYFP showed obvious nuclear localization, whereas aa18-31-EYFP showed pan-cellular distribution, with the exception of nucleolus, suggesting aa 1 to 17 contains functional NLS, which may be executed by NLS1.

To characterize the minimum NLS region, aa 1 to 17 was also cut into two segments aa 1 to 8 and aa 9 to 17 (NLS1), then they were tagged with EYFP (aa1-8-EYFP and aa9-17-EYFP) (Figure 3A) and tranfected into COS-7 cells. As results (Figure 3B), the nuclear fluorescence of aa9-17-EYFP only showed a slight more than that of the cytoplasm, indicating deletion of aa 1 to 8 significantly alters the nuclear accumulation of aa 9 to 17, although aa1-8-EYFP showed pan-cellular localization.

To further confirm aa 1 to 17 was a genuine functional NLS, this fragment was fused with a completely cytoplasmic protein, EYFP-ECRF4 (~60kDa) [24], to generate pUL2(NLS)-EYFP-ECRF4, which was then transfected into COS-7 cells to detect its subcellular localization. As shown in Figure 3C, EYFP-ECRF4 showed evidently cytoplasmic distribution, while the pUL2(NLS)-EYFP-ECRF4 fusion protein could noticeably import into the nucleus under the cooperation of UL2 NLS, suggesting UL2 NLS can translocate the cytoplasmic protein into the nucleus. These results undoubtedly uncovered that the vital region that responsible for the nuclear localization of UL2 was profiled to aa 1 to 17, and the arginine-rich motif of ^12^RRR^14^ may be very important and indispensable for the nuclear trafficking of UL2.

To further verify this deduction, arginine residues within the ^12^RRR^14^ sequence of full-length UL2 was replaced with neutral alanine residues to produce ^12^AAA^14^, and fused with EYFP to yield pUL2(12-14)mut-EYFP (Figure 3A). As expected, replacement of ^12^RRR^14^ abrogated the exclusively nuclear localization of UL2 [pUL2(12-14)mut-EYFP] (Figure 3D). Therefore, these evidences indicated that the basic-rich region in NLS1 is essential for UL2 nuclear transport, and the functional NLS of UL2 is a 17-residue peptide ^1^MKRACSRSPSPRRRPSS^17^.

### Characterization of the functional NES of UL2

**Figure 4:**
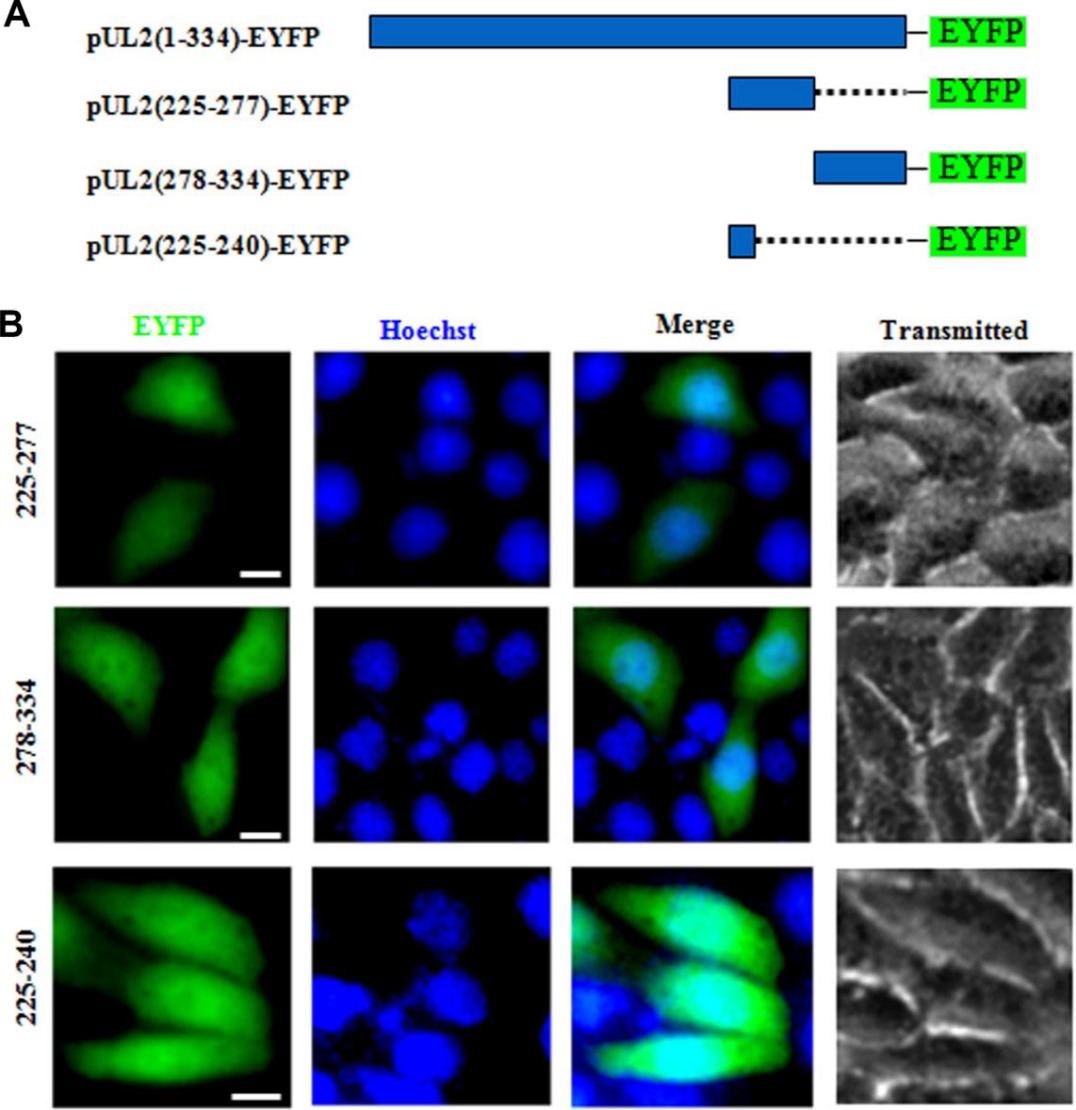
**Determination the presμmed NES function of UL2.** (**A**) Schematic representation of WT UL2 and its deletion mutants UL2(225-277), UL2(278-334) and UL2(225-240) fused with EYFP. (**B**) Subcellular localization of these UL2 deletion mutants (shown in **A**) in plasmid-transfected live COS-7 cells. All scale bars indicate 10 μm.

NESs are reported to be predominantly composed of hydrophobic, leucine-rich sequences [29], which are crucial for the nuclear export [28]. As mentioned above, the primarily cytoplasmic accumulation of aa225-334 (Figure 1B) suggested that UL2 may possess a functional NES. Bioinformatics analysis of NetNES 1.1 demonstrated that UL2 contains some leucine-rich motifs, particularly aa 225 to 240 (LEKWARDGVLLLNTTL) (Figure 1C, red color letters), which was designated as NES. In an endeavor to further diagram the NES of UL2, three deletion mutants encompassing aa 225 to 277, aa 278 to 334 and aa 225 to 240 fused with EYFP (aa225-277-EYFP, aa278-334-EYFP and aa225-240-EYFP) were constructed (Figure 4A) and tested in COS-7 cells. As shown in Figure 4B, all of them showed similar subcellular distribution pattern to that of aa1-8-EYFP, with pan-cellular localization, indicating the predicted NES is nonfunctional, and the functional NES of UL2 may be formed by the space conformation.

Taken together, by creating a series of deletion mutants fused with EYFP and fluorescence microscopy analysis, a N-terminus basic aa rich NLS (^1^MKRACSRSPSPRRRPSS^17^) (encompassing key aa ^12^RRR^14^) of HSV-1 UL2 was characterized, whereas its predicted NES is nonfunctional.

### BACs construction of UL2 related recombinant virus and virus rescue

**Figure 5:**
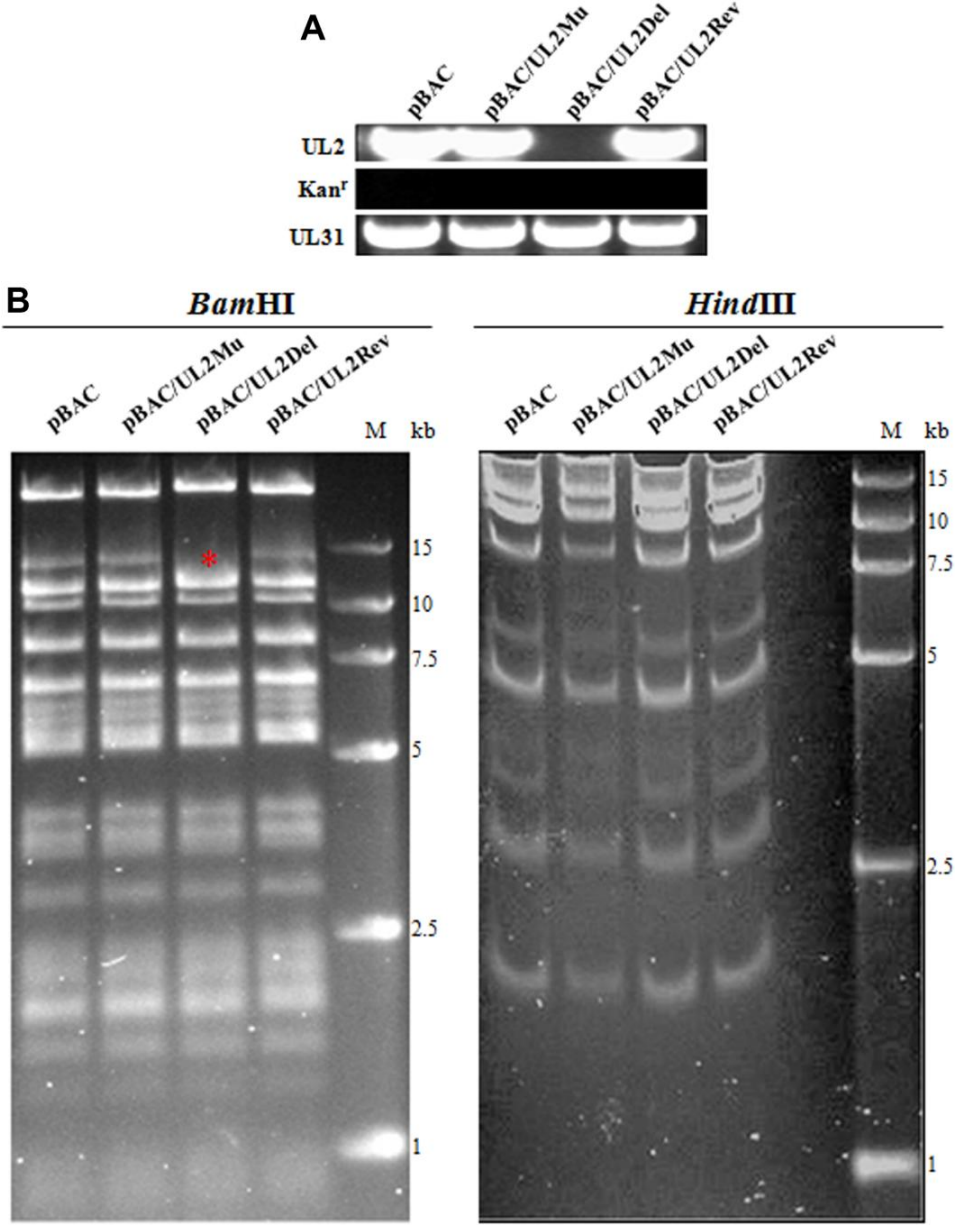
**BACs construction of UL2-related recombinant HSV-1.** (**A**) PCR analysis of the constructed recombinant BACs. The UL2, UL31 and Kan^r^ genes were amplified from WT pBAC (lane 1), pBAC/UL2Mu (lane 2), pBAC/UL2Del (lane 3) and pBAC/UL2Rev (lane 4), respectively. (**B**) Gel electrophoresis (0.8%) of WT pBAC (lane 1) and recombinant BACs pBAC/UL2Mu (lane 2), pBAC/UL2Del (lane 3) and pBAC/UL2Rev (lane 4) analyzed by *Bam*HI *and Hind*III restriction digestion, respectively. The red asterisk indicates the specific band that was disappeared only in pBAC/UL2Del genome when all the BACs were treated with *Bam*HI digestion. Marker sizes in kb are indicated on the right side of the gels.

In order to further investigate the effect of UL2 subcellular localization on the growth of HSV-1, UL2 related recombinant virus BACs were constructed based on the parental BAC (wild type (WT) HSV-1 BAC GFP Luc, named pBAC) [30] and two-step Red-mediated homologous recombination technique, which allows for scar-free mutation, deletion and insertion in the target sequence (see details in *Materials and Methods*) [31]. The HSV-1 BAC GFP Luc/UL2 deletion (pBAC/UL2Del) was firstly constructed, then HSV-1 BAC GFP Luc/UL2(12-14) Mut (pBAC/UL2Mu) and HSV-1 BAC GFP Luc/UL2 revertant (pBAC/UL2Rev) were constructed based on pBAC/UL2Del, and the expected recombinant clones were eventually selected with chloramphenicol resistance. As results, PCR analysis (Figure 5A) and sequencing (data not shown) showed that each clone obtained the expected mutation. Restriction fragment length polymorphism analysis of pBAC/UL2Del, pBAC/UL2Mu and pBAC/UL2Rev showed similar patterns to that of the parental pBAC when all the BACs were treated with *Hind*III digestion, whereas specific band of only pBAC/UL2Del disappeared when all the BACs were treated with *Bam*HI digestion (Figure 5B, red asterisk), suggesting the appropriate recombination occurred in the expected position, and the UL2 related recombinant BACs were successfully constructed. To rescue the UL2 related recombinant viruses, diverse UL2 related recombinant BACs DNA were transfected into Vero cells, and results showed that the recombinant viruses vUL2, vUL2Del, vUL2Mu and vUL2Rev (produce visible GFP) were successfully rescued from pBAC, pBAC/UL2Del, pBAC/UL2Mu and pBAC/UL2Rev, respectively (data not shown).

### Subcellular localization of UL2 in recombinant virus-infected cells

**Figure 6:**
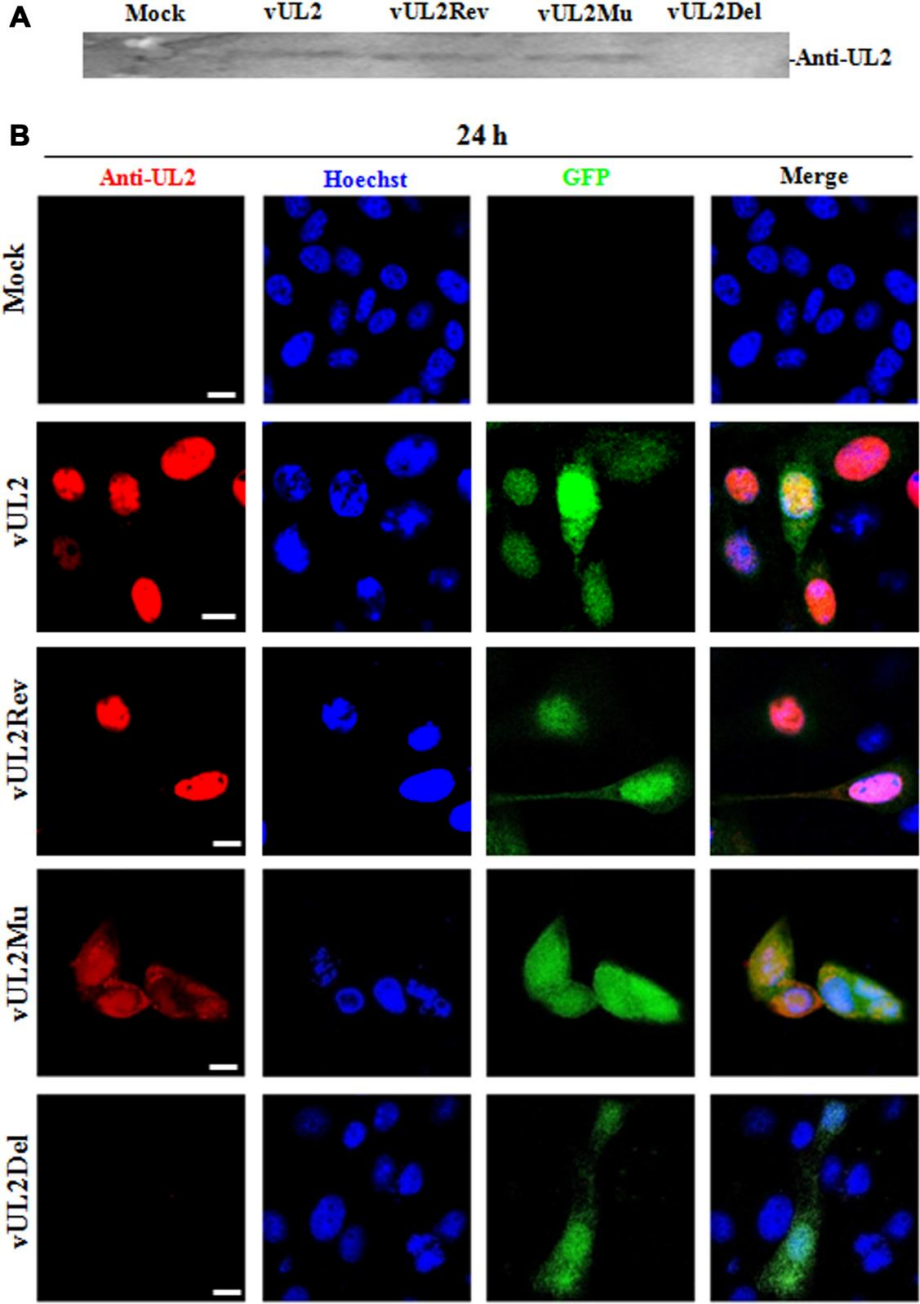
**Protein expression and subcellular localization of UL2 in different recombinant viruses-infected cells.** (**A**) Monolayer HEK293T cells were infected with different reconstitute virus vUL2, vUL2Del, vUL2Mu or vUL2Rev at an MOI of 1 for 24 h, and cells were harvested when CPE reached 90-95%. Cell lysates were then subjected to WB analysis using the prepared anti-UL2 pAb and AP-conjugated goat anti-rat IgG. (**B**) Vero cells infected with different reconstitute virus vUL2, vUL2Del, vUL2Mu or vUL2Rev at an MOI of 1 for 24 h, then cells were subjected to IFA using anti-UL2 pAb and Dylight 649 conjugated goat anti-rat IgG, to show the subcellular localization of UL2. Cells were finally counterstained with Hoechst to visualize the nuclei. GFP was also captured to show the cells were successfully infected by HSV-1. All scale bars indicate 10 μm.

To confirm the expression of UL2 mutants from these reconstituted viruses, HEK293T cells infected with different recombinant viruses were lysed for western blot (WB) analysis by using our prepared anti-UL2 polyclonal antibody (pAb). As expected, the pAb could specifically detect a target band about 37-kDa (UL2) in vUL2-, vUL2Mu- and vUL2Rev-infected cells, but not in the mock-infected or vUL2Del-infected cells (Figure 6A). In order to verify whether the NLS of UL2 is functional in the course HSV-1 infection, indirect immunofluorescence analysis (IFA) was performed to probe the subcellular localization of UL2 and its mutants in Vero cells infected with these reconstituted viruses. As results, UL2 could be detected in the nucleus during vUL2 and vUL2Rev infection (Figure 6B), whereas UL2 harboring NLS mutation showed pan-cellular distribution during vUL2Mu infection, which was similar to the subcellular localization pattern of UL2(12-14)mut-EYFP in transfected COS-7 cells (Figure 3B). These results showed that the identified NLS can play a nuclear accumulation role for UL2 during HSV-1 infection.

### Efficient production of HSV-1 requires the nuclear targeting of UL2

**Figure 7:**
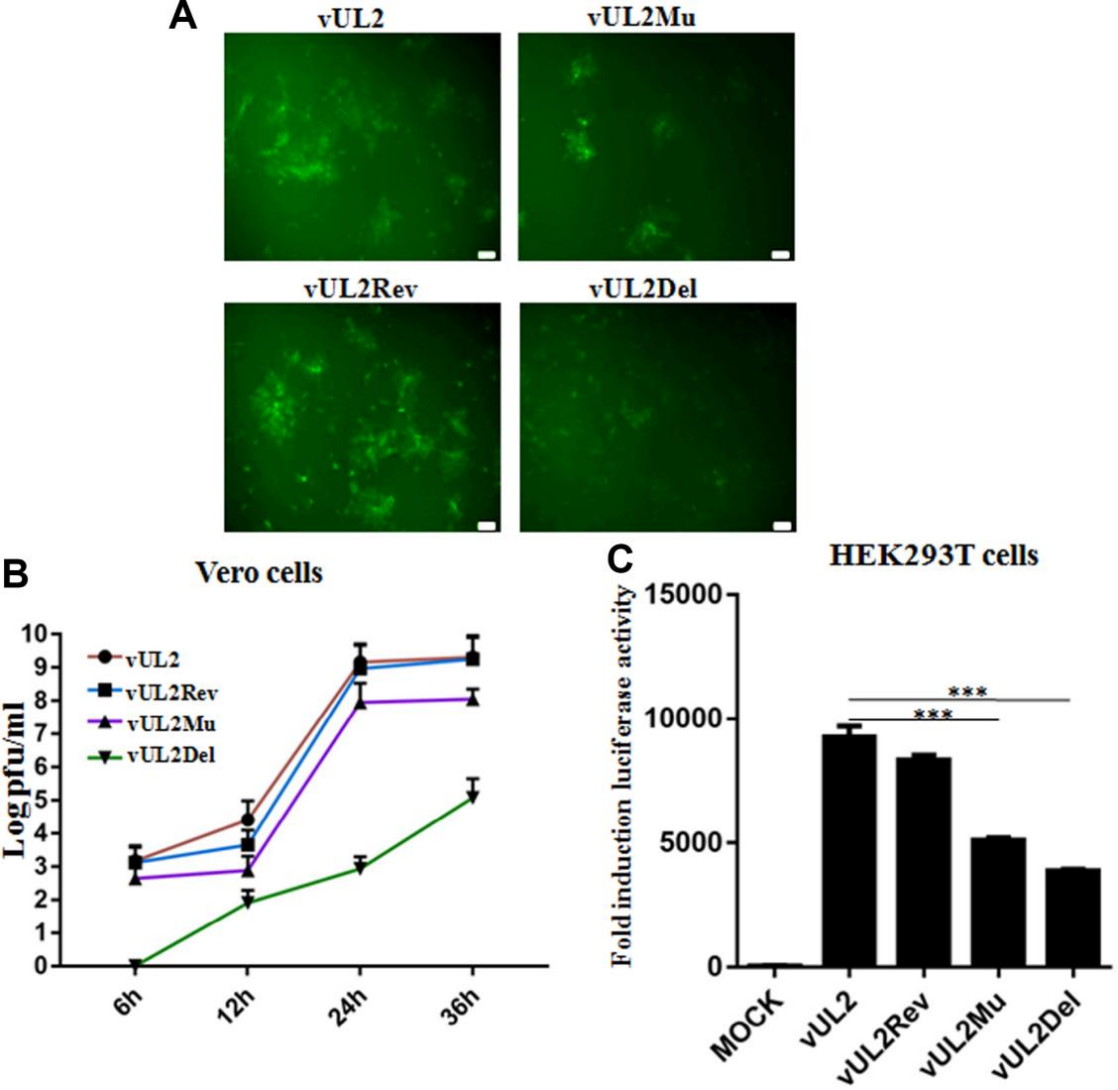
**Nuclear targeting of UL2 is important for efficient HSV-1 production.** (**A**) Plaque analysis of WT HSV-1 (vUL2) and its derived recombinant viruses (vUL2Del, vUL2Mu and vUL2Rev) by live cells fluorescence microscope. Confluent Vero cells were infected with the indicated viruses at an MOI of 1. After adsorption at 37°C for 2 h, virus was washed away and the plate was covered with DMEM-2% FBS, then the fluorescences (GFP) derived from these viruses were analyze by fluorescence microscope after infection for 24 h. (**B**) Growth curve analysis of WT HSV-1 and its derived recombinant viruses. Vero cells were infected with the indicated viruses at an MOI of 1 for 6, 12, 24 and 36 h, then virus was harvested, and their titers were determined on the Vero monolayer by plaque method (with crystal violet staining). The data shown was the average results from three independent experiments. (**C**) Luciferase activity was used to determine the viral replication of WT HSV-1 and its derived recombinant viruses in HEK293T cells. HEK293T cells were infected with the indicated viruses at an MOI of 1 for 24 h, then luciferase activity was detected by harvesting the lysates of the virus-infected HEK293T cells. Data were expressed as means ± SD from three independent experiments. Statistical analysis was performed using student’s *t* test, and *** indicates *P* < 0.001. All scale bars indicate 30 μm.

To further probe whether the nuclear targeting of UL2 influences the viral replication of HSV-1, stocks of WT HSV-1 (vUL2) and recombinant viruses (vUL2Del, vUL2Mu and vUL2Rev) were prepared and their titers were determined, then the plaque formation and viral propagation property of each recombinant virus were tested at an multiplicity of infection (MOI) of 1. Although vUL2Del was visible, vUL2Del-induced plaques formation appeared later and less than that of vUL2, while vUL2Mu-induced plaques showed moderate decrease. However, the plaque phenotype of vUL2Rev reverted to vUL2 when UL2 allele was repaired (Figure 7A), suggesting the NLS of UL2 is important for the plaque formation. In order to explore the molecular mechanism of different plaque phenotypes caused by diverse recombinant viruses, the growth kinetics of these viruses were measured when Vero cells were infected (MOI=1) with these viruses and then harvested at the indicated time points (Figure 7B). As results, the growth kinetics of vUL2Rev was comparable to that of vUL2. However, the growth kinetics of vUL2Del was apparently slower than that of parental virus vUL2 at all the tested times, and NLS mutation of UL2 also could obviously reduce the growth kinetics of vUL2Mu (Figure 7B). Besides, luciferase activity assay was used to analyze the viral replication of these reconstituted recombinant viruses in HEK293T cells. Compared with the replication dynamics of vUL2 and vUL2Rev, replication of vUL2Del decreased significantly, and the replication kinetics of vUL2Mu was close to that of vUL2Del (Figure 7C). Collectively, these results showed that the nuclear targeting of UL2 mediated by its NLS is important for the efficient production of HSV-1.

### Nuclear translocation of UL2 is necessary for effective viral DNA replication and gene transcription

**Figure 8:**
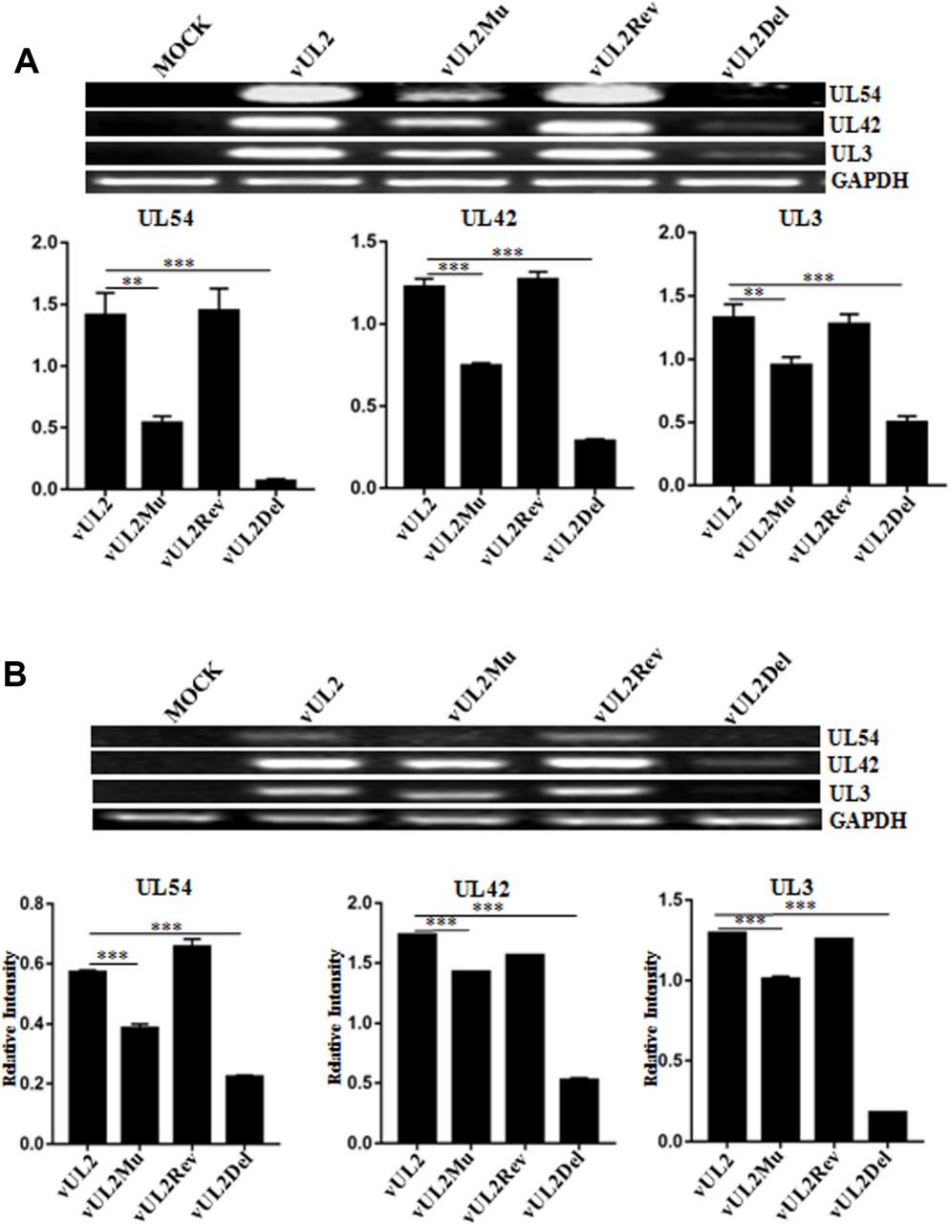
**Viral DNA replication and mRNA expression analysis of WT HSV-1 and its derived recombinant viruses**. (**A**) DNA replication analysis of WT HSV-1 and its derived recombinant viruses. HEK293T cells were mock-infected or infected with WT HSV-1 (vUL2) and its derived recombinant viruses (vUL2Del, vUL2Mu and vUL2Rev) at an MOI of 1 for 24 h. Then, total cellular DNA was purified and PCR was performed with the primers specific for UL54 (IE gene), UL42 (E gene) and UL3 (L gene) to quantify DNA levels. To ensure that an equal amount of DNA was used from each sample, the DNA of each sample was normalized with GAPDH. (**B**) mRNA expression analysis of WT HSV-1 and its derived recombinant viruses. HEK293T cells were mock-infected or infected with WT HSV-1 (vUL2) and its derived recombinant viruses (vUL2Del, vUL2Mu and vUL2Rev) at an MOI of 1 for 24 h. Then, total RNA was isolated, and the mRNA expression levels of UL54, UL42, UL3 and GAPDH were assessed by RT-PCR. GAPDH was served as an internal control. Densitometry of UL54, UL42 and UL3 bands were normalized to the control GAPDH. Data were expressed as means ± SD from three independent experiments. Statistical analysis was performed using student’s t test, and * indicates *P* < 0.05, ** indicates *P* < 0.01, *** indicates *P* < 0.001.

To continue dissect the effect of UL2 NLS on the DNA replication of HSV-1 genes from diverse phases, total DNA of the reconstitute virus-infected (MOI=1) cells was extracted, then the representatives of immediate early (IE) gene (UL54), early (E) gene (UL42), late (L) gene (UL3) and GAPDH gene were amplified by PCR. Compared with the effect of vUL2Del, mutation of UL2 (vUL2Mu) also remarkably diminished viral DNA replication (Figure 8A), suggesting efficient viral DNA replication requires UL2 expression and its nuclear targeting. To further examine the impact of UL2 NLS on the mRNA expression of HSV-1 genes from different phases, total RNA of the reconstitute virus-infected (MOI=1) cells was isolated, and the mRNA levels of UL54, UL42, UL3 and GAPDH were detected by RT-PCR. Consistent with the aforementioned result, mRNA expression of all the detected genes was notably lessen in vUL2Mu-infected cells when compared with that of the vUL2-infected cells (Figure 8B). Consequently, these data suggested that the NLS- mediated nuclear transport of UL2 is important for efficient viral DNA replication and gene transcription.

## DISCUSSION

It’s well known that characterization of the subcellular localization is a favorable way to assess the potential roles of a large number of proteins [32]. In our previous study, we found that in the HSV-1 encoded proteins, 21 proteins show cytoplasmic or subcytoplasmic localization, 16 proteins demonstrate nuclear or subnuclear distribution, and other proteins exist in both the nucleus and cytoplasm [14]. Furthermore, most of envelope proteins show cytoplasmic localization, while most of capsid proteins appear to be enriched or completely localized in the nucleus, suggesting the subcellular distribution of a specific protein is associated with its function execution during viral replication [14].

Our previous study showed that UL2 is located exclusively in the nucleus without the presence of other viral constituents, which is mediated under the assistance of different nuclear import receptors [15]. In this study, we continued to identify the functional NLS of UL2 and assess its effect of nuclear targeting during HSV-1 infection. Bioinformatics analysis showed that UL2 contains two potential NLSs in the arginine-rich regions (aa 9 to 17 and aa 69 to 75) and one leucine-rich motif (aa 225 to 240). By constructing a series of truncated mutants of UL2 fused with EYFP, the *bona fide* functional NLS was identify at aa 1 to 17, with the key aa ^12^RRR^14^ that is essential for the nuclear accumulation of UL2. However, the predicted NES was nonfunctional, which may probably correlate with the space conformation of UL2.

Herpesviral UDG is reported to be a multi-functional protein, which is highly conserved and important for the production of viral DNA among HSV-1 UL2, HSV-2 UL2, pseudorabies virus (PRV) UL2, HCMV UL114, varicella-zoster virus ORF59 and human herpesvirus 6 U81. Specifically, UDG is involved in the base cutting repair pathway, which correctly detaches the erroneously inserted uracil from synthetic viral DNA [5, 8, 33]. Studies have shown that the NLSs of some viral proteins are required for the efficient virus replication. For example, basic aa-constituted N-terminus NLS of VP1-2 is favorable for HSV-1 replication and gene expression [34], NLS of UL31 is important for PRV replication [35], and the NLS- mediated nuclear targeting of HCMV large tegument protein UL48 is essential for viral growth [36]. To further investigate the role of the identified NLS-mediated nuclear transport of UL2 during viral replication, HSV-1 mutants with UL2 deletion (vUL2Del), arginine-rich motif ^12^RRR^14^ mutation (vUL2Mu) and UL2 reversion (vUL2Rev) were constructed.

Compared with vUL2Del-infected cells, vUL2Mu also could obviously decrease the growth kinetics of viral replication (Figure 7B and 7C), indicating the nuclear translocation of UL2 is important for efficient HSV-1 replication. Moreover, the NLS-mediated nuclear import of UL2 also could prominently affect viral DNA replication (Figure 8A) and mRNA accumulation (Figure 8B). Therefore, NLS-mediated nuclear targeting of UL2 is important for HSV-1 replication.

Taken together, this study will not only benefit us to expand our knowledge about the biological function of UL2 and HSV-1 pathogenesis, but also offer a theoretical basis for the further design of new antiviral drug target and development of qualified vaccine against HSV-1.

## MATERIALS AND METHODS

### Cells and virus

Human embryonic kidney (HEK) 293T, COS-7 and Vero cells were cultured at 37 °C in Dulbecco’s modified MEM (DMEM, Gibco-BRL) added with 10% heat inactivated fetal bovine serum (FBS, Gibco-BRL). WT HSV-1 (F strain) BAC GFP Luc (simultaneously expressing firefly luciferase and GFP tag) was a generous gift from Dr. Chunfu Zheng (School of Basic Medical Sciences, Fujian Medical University) [30].

### Antibodies

Dylight 649 conjugated goat anti-rat IgG was obtained from Abbkine. Alkaline phosphatase (AP)-conjugated goat anti-rat IgG was provided by Affinity Biosciences. Anti-UL2 pAb was prepared in rat (unpublished data) and stored in our lab.

### Plasmids construction

The enzymes used for molecular cloning were obtained from Thermo Scientific except Ex Taq DNA polymerase from TaKaRa and T4 DNA Ligase from Invitrogen. Plasmid pUL2-EYFP was constructed in our previous study [15]. UL2 deletion mutants (including aa substitution) were yielded by PCR-ligation-PCR mutagenesis [21–23] using proper primers (sequences available upon request), then the PCR fragment was inserted into pEYFP-N1 (Clontech), as described previously [16, 18, 19, 37, 38]. pUL2(NLS)-EYFP-ECRF4 was constructed by inserting the oligonucleotides of UL2 NLS into the *B*glII and *H*indIII digested pEYFP-ECRF4. All the constructed plasmids were validated by PCR, restriction analysis and sequencing.

### Plasmid transfection and fluorescence microscopy

To analyze the subcellular distribution of recombinant UL2 proteins in live cells, plasmid transfection and fluorescence microscopy assays were carried out as reported in our previous studies [15, 17, 20–23, 39]. Briefly, COS-7 cells were cultured in DMEM added with 10% FBS overnight to reach the confluency 60-80% before transfection. The next day, monolayer cells were transfected with indicated plasmid DNA mixed with polyethylenimine (Polysciences) according to the manufacturer’s instructions. 24 h post-transfection, cells were subjected to fluorescence microscopy analysis, which were tested using a OLYMPUS fluorescence microscope (IX71, objective lens LUCPlanFLN 40×/NA 0.60, Olympus Optical Co., Tokyo, Japan). All the pictures were captured with an enlargement of 400×, and each picture represents most of the cells with homologous subcellular localization. Light-translucent pictures are introduced to show cellular morphology. Cells were stained with Hoechst 33342 (Beyotime) to visualize the nuclei. In the same observation, each transfection was repeated for three times, and data presented were from one illustrative experiment. Fluorescent images of EYFP fusion proteins were shown in pseudocolor green, and images were processed using Adobe Photoshop. All scale bars indicate 10 μm.

### Construction of recombinant HSV-1 BAC

Recombinant HSV-1 BAC was constructed using two-step Red-mediated recombination method, as described previously [25, 40]. In the first step of Red recombination, a kanamycin resistance gene expression cassette (kan^r^) flanked with homology arms of partial coding sequences of UL1 and UL3 was amplified from the plasmid pEPkan-S (provided by Dr. Nikolaus Osterrieder, Department of Microbiology and Immunology, College of Veterinary Medicine, Cornell University) by PCR using DNA polymerase KOD FX (TOYOBO) [25, 40]. The obtained fragment was digested with DpnI (New England Lab) and purified using a gel extraction kit (TIANGEN), then electroporation transformed into *Escherichia coli* GS1783 (provided by Dr. Gregory A. Smith, Department of Microbiology-Immunology, Northwestern University) [31] harboring WT HSV-1 BAC GFP Luc. Subsequently, recombinants were plated on a plate (containing chloramphenicol and kanamycin) at 32°C bacterial incubator overnight. Next day, one positive clone named WT HSV-1 BAC GFP Luc/kan was selected for PCR verification and sequencing analysis, which was then used for the second step of Red recombination.

The second step of homologous recombination was performed to generate recombinant BAC with UL2 deletion, revertant or NLS mutation [UL2(12-14)Mut]. The kan^r^ cassette of WT HSV-1 BAC GFP Luc/kan was deleted (UL2 deletion) or replaced by WT UL2 gene (UL2 revertant) or UL2(12-14)Mut fragment when GS1783 was treated with arabinose at 42 °C [25], then bacteria were subjected to resistance screening on kanamycin-containing plate or chloramphenicol-containing plate, and recombinant bacteria only grown on the chloramphenicol plate (but not kanamycin plate) were identified by colony PCR, which were then extracted for the recombinant BACs DNA [HSV-1 BAC GFP Luc/UL2 deletion, HSV-1 BAC GFP Luc/UL2(12-14)Mut and HSV-1 BAC GFP Luc/UL2 revertant, which were shorted for pBAC/UL2Del, pBAC/UL2Mu and pBAC/UL2Rev, respectively] for further PCR analysis, restriction fragment length polymorphism (RFLP) analysis and sequencing analysis.

### Recombinant virus rescue

To reconstitute recombinant viruses from the mentioned HSV-1 BACs DNA, including pBAC, pBAC/UL2Del, pBAC/UL2Mu and pBAC/UL2Rev (encoding for viruses vUL2, vUL2Del, vUL2Mu and vUL2Rev, respectively), the polyetherimide transfection reagent was used to transfect with 2μg of the corresponding BAC DNA into Vero cells. After transfection, virus was harvested when the cytopathic effect achieves 90 to 95%. Then, the BAC-transfected cell lysates were collected and inoculated on monolayer of Vero cells cultured in 10-cm-diameter dish for 3 to 4 consecutive serial passages to increase virus titers.

### WB analysis

WB analysis was performed as previously described [37, 38, 41]. Briefly, HEK293T cells were mock-infected or infected with various viruses vUL2, vUL2Del, vUL2Mu or vUL2Rev at an MOI of 1 for 24 h, then cell lysates were collected, separated by 10% SDS-PAGE and transferred to the nitrocellulose membrane (Pall Corporation). The membrane was then blocked with 5% skim milk and incubated overnight at 4 °C with the prepared anti-UL2 pAb (1:500 dilution). After washing 3 times with Tris-buffered saline with Tween-20 (TBST), the membrane was incubated with AP-conjugated goat anti-rat IgG (1:2,000 dilution) at 37 °C for 1 h. Protein bands were then detected by nitroblue tetrazolium (NBT)/5-bromo-4-chloro-3-indolylphosphate (BCIP) (BIOSHARP) and finally terminated by distilled water.

### IFA

To investigate the subcellular localization of UL2 in HSV-1-infected cells, IFA was performed as described previously [26, 37–39, 42]. In short, Vero cells were infected or mock-infected with the reconstituted virus vUL2, vUL2Del, vUL2Mu or vUL2Rev at an MOI of 1 for 24 h, then cells were subjected to IFA using anti-UL2 pAb as primary antibody and Dylight 649 conjugated goat anti-rat IgG as secondary antibody. After incubating with related antibody, cells were washed with PBS, and the cell nucleus were stained with Hoechst 33342. Cells were analyzed by Leica SP8 confocal microscope using 400× oil-immersion objective. All scale bars indicate 10 μm.

### Plaque assays and growth curve analysis

Vero cells were infected with the reconstituted virus vUL2, vUL2Del, vUL2Mu or vUL2Rev at an MOI of 1. After 2 h of adsorption at 37°C, virus dilutions were washed off, and the culture plate was overlaid with DMEM-2% FBS and white agar (1:1). After incubation at 37°C for 24 h, the cell monolayer was fixed with methanol. Then, the plaque phenotype of different reconstituted viruses (with GFP marker) was observed by fluorescence microscopy assays.

For analyzing the growth curve, Vero cells were infected with various reconstituted viruses at an MOI of 1. After adsorption for 2 h at 37 °C, the virus was discarded, and the culture plate was covered with DMEM-2% FBS for 6, 12, 24 and 36 h. Then, viruses were harvested and their titers were determined by plaque method with crystal violet (MACKUN) staining. Viral titers in all samples were determined in triplicate on monolayer Vero cells, and the respective mean values were shown.

### Luciferase analysis

The luciferase analysis was performed as described previously [39, 41, 43]. In short, HEK293T cells cultured in 12-well plate (Corning) were infected with the virus vUL2, vUL2Del, vUL2Mu or vUL2Rev at an MOI of 1. 24 h post-infection, infected cells were lysed with RIPA lysis buffer (Beyotime Biotechnology, 50 mM Tris-HCl, pH 7.5, 150 mM NaCl, 1% Triton X-100, 2 mM EDTA, 1 mM sodium orthovanadate, 1 mM phenylmethanesulfonyl-fluoride, 10 μg/mL aprotinin, and 10 μg/mL leupeptin) and harvested, then luciferase activity was detected using a luciferase assay kit (Promega). Data were expressed as means ± standard deviations (SD) from three independent experiments.

### Viral DNA isolation and analysis

To measure whether viral DNA replication was affected in the case of different viruses infection, HEK293T cells cultured in 6-well plate were infected with vUL2, vUL2Del, vUL2Mu or vUL2Rev virus at an MOI of 1. 16 h post-infection, the infected cells were harvested and lysed with lysis buffer (0.5% sodium dodecyl sulfate [SDS], 50 mM Tris-HCl [pH 7.4], 100 mM NaCl, 0.1 g/ml proteinase K, 25 g/ml RNase A and 5 mM EDTA) at 50 °C for overnight. Subsequently, cell lysate was treated twice with an equal volume of isoamyl alcohol/chloroform/phenol (1:24:25) and once with isoamyl alcohol/chloroform (1:24). The DNA was then precipitated with isopropanol at room temperature and resolved in Tris-EDTA buffer. Finally, the extracted DNA was analyzed by PCR using IE gene UL54 (F: 5′-ATGGCGACTGACATTGATATG-3′, R: 5′-AAACA GGGAGTTGCAATAAAAAT-3′), E gene UL42 (F: 5′-ATGACGGATTCCCCTGGC-3′, R: 5′-GGGGAAT CCAAAACCAGAC-3′), L gene UL3 (F: 5′-ATGGTT AAACCTCTGGTCTC-3′, R: 5′-CTCGGCCCCCGAG GCCAG-3′) and GAPDH (F: 5′-AGGTCGGTGTGAA CGGATTTG-3′, R: 5′-TGTAGACCATGTAGTTGA GGTCA-3′) specific primers. Densitometric analysis of the bands was performed using Image J software (National Institutes of Health, Bethesda, MD, USA), and statistical analysis of densitometric data was carried out by student’s *t* test.

### RNA isolation and semiquantitative reverse transcription PCR

To analyse the mRNA expression of HSV-1 genes from different phases, reverse transcription PCR (RT-PCR) was employed. HEK293T cells were mock-infected or infected with vUL2, vUL2Del, vUL2Mu or vUL2Rev virus at an MOI of 1. 16 h post-infection, total RNA was extracted by TRIzol (Invitrogen) according to the manufacturer's instructions. Then, extracted RNA was reverse transcribed to cDNA using a reverse transcription kit (GenStar). Using the obtained cDNA as a template, PCR analysis was performed using specific primers for UL54 (F: 5′-TTGGTCCTGCGCT CCATCTC-3′, R: 5′-GTCTGGTCTCGGCGTCAAAG -3′), UL42 (F: 5′-GAGTACCTGCGTCACATTTG-3′, R: 5′- GTCGTGAGGAAGAACTTGAG-3′), UL3 (F: 5′-AATTAGGGCGTCGCCAGCTC-3′, R: 5′-AAC GACCCGAAGCTGCTCTC-3′) and GAPDH (F: 5′-AGGTCGGTGTGAACGGATTTG-3′, R: 5′-TGTAG ACCATGTAGTTGAGGTCA-3′). Finally, the PCR product was analyzed on a 2% agarose gel. Densitometric analysis of the bands was performed using Image J software, and statistical analysis of densitometric data was carried out by student’s *t* test.
